# Factors mediating the psychological well-being of healthcare workers responding to global pandemics: A systematic review

**DOI:** 10.1177/13591053211012759

**Published:** 2021-04-29

**Authors:** Jekaterina Schneider, Deborah Talamonti, Benjamin Gibson, Mark Forshaw

**Affiliations:** 1 University of Jyväskylä, Finland; 2 Research centre and Centre EPIC, Montreal Heart Institute, Canada; 3 Liverpool John Moores University, UK

**Keywords:** COVID-19, healthcare professionals, mediation, mental health, pandemic

## Abstract

This paper reviewed mediators of psychological well-being among healthcare workers responding to pandemics. After registration on PROSPERO, a systematic review was performed in four databases and 39 studies were included. Worse mental health outcomes, such as stress, depressive symptoms, anxiety and burnout were related to demographic characteristics, contact with infected patients and poor perceived support. Self-efficacy, coping ability, altruism and organisational support were protective factors. Despite limitations in the quality of available evidence, this review highlights the prevalence of poor mental health in healthcare workers and proposes target mediators for future interventions.

## Introduction

The novel coronavirus (COVID-19) outbreak, caused by infection with severe acute respiratory syndrome coronavirus-2 (SARs-CoV-2), received pandemic status by the World Health Organization (WHO) in March 2020 (Cucinotta and Vanelli, 2020). Pandemics are defined by the WHO as the worldwide spread of a new disease (WHO, 2020a) and the term has been used to describe outbreaks of similar coronavirus diseases, such as severe acute respiratory syndrome (SARS) and Middle East respiratory syndrome (MERS), as well as the outbreaks of influenza (H1N1), Swine flu (H1N1/09) and Ebola. Pandemics are also at least partly categorised by their rapid incidence and a consequence of this is that they often put high pressure on healthcare workers (HCWs) and healthcare service capacities. COVID-19 in particular has a high transmission rate, which means that, despite a relatively low mortality rate of 2%, the virus’ associated mortality is higher than that of SARS and MERS combined (Mahase, 2020). Pandemics, therefore, place massive burdens not only on the physical and mental health of the general population (e.g. Vinkers et al., 2020), but also on the HCWs who play key roles during such events (Cullen et al., 2020). Admittedly, however, less is known about their impact on countries across Europe and North America, which have been considerably less affected in recent history.

Research has consistently shown that individuals in healthcare professions experience higher levels of work-related stress, burnout and psychological ill-health than the general population, even under ‘normal’ circumstances (Hofmann, 2018), and are reluctant to seek help due to fear of stigma and detrimental effects on future career prospects (Chew-Graham et al., 2003). During acute health crises, such as COVID-19 and other infectious disease outbreaks, these issues may be further exacerbated. Health professionals, especially those working in direct contact with suspected or confirmed patients with infectious diseases, may experience stigmatisation as a result of their job, fear of contagion, fear of spreading the disease to others and feelings of isolation if they have to be quarantined or separated from their loved ones on account of their exposure to high viral loads. Some possible reasons for the adverse psychological outcomes seen in HCWs during health emergencies stem from increased workload and/or work hours, inadequate personal protective equipment (PPE), being overexposed to pandemic reports in the media, experiencing a high rate of infection and feeling inadequately supported by their employer or organisational structure (Cai et al., 2020; Devnani, 2012; Lee et al., 2018; Lietz et al., 2016; Styra et al., 2008; Tam et al., 2004). As HCWs are considered essential workers during outbreaks of infectious diseases, protecting their psychological well-being is a priority (Arden and Chilcot, 2020; Bao et al., 2020; Chen et al., 2020; Galbraith et al., 2021; Holmes et al., 2020; Xiang et al., 2020). Specifically, more information is required on the protective and risk factors that influence the psychological well-being of HCWs responding to global pandemics.

Previous reviews have been conducted to explore the mental health of HCWs during infectious disease outbreaks. Two recent reviews found a high prevalence of stress, anxiety, depression and insomnia among HCWs during the current COVID-19 outbreak (Pappa et al., 2020; Spoorthy, 2020). Other reviews on the mental health of HCWs during infectious disease outbreaks or following a disaster found that compared with lower risk controls, high-risk HCWs had greater levels of post-traumatic stress, psychological distress and depressive symptoms (e.g. Kisely et al., 2020; Naushad et al., 2019). Several of these reviews have also identified various protective and risk factors associated with psychological distress in HCWs. The most commonly reported protective factors included clear communication, social support, practical support (e.g. the provision of appropriate work attire and access to adequate PPE) and getting sufficient rest. The most commonly reported risk factors included exposure to infected patients, being younger or less experienced, knowing someone who has been infected/having an infected family member, being quarantined, lack of practical and social support and experiencing stigma (e.g. Arora and Grey, 2020; Koh et al., 2005; Maunder et al., 2004; Tam et al., 2004). However, at present, the majority of studies are of low quality due to high risk of bias (e.g. limitations in study design, recall bias, selection bias) and imprecise results (De Brier et al., 2020). Additionally, few studies have so far conducted formal mediation analyses on protective and risk factors that go beyond mere association to identify possible mediators of psychological well-being of HCWs responding to global pandemics.

To our knowledge, no systematic review has so far considered all recent global pandemics to identify recurrent mediators of psychological well-being in HCWs. Instead, previous reviews have been limited to COVID-19 or coronaviruses, which might exclude important data and wider patterns, or have been limited in their methodology, with little consideration of risk and protective factors or the quality of the research reviewed. As such, effective strategies for supporting the mental health and well-being of HCWs in the context of pandemics are currently unclear (Li et al., 2020; Zhang et al., 2020b). Therefore, we performed a systematic review to identify the mediators of psychological well-being in HCWs responding to global pandemics. The findings from this review will provide evidence for the potential mechanisms that can be targeted by interventions to protect HCWs’ mental health and psychological well-being in the current context of the ongoing COVID-19 pandemic and in future emergencies.

## Methods

This review was conducted according to the Preferred Reporting Items for Systematic Reviews and Meta-Analyses statement (PRISMA; Moher et al., 2010) and pre-registered on PROSPERO (ref. CRD42020187340).

### Data sources and search strategies

A systematic search was conducted for papers published up to 7 June 2020 using the databases Google Scholar, PsycINFO, MEDLINE (PubMed) and Web of Science. Boolean combinations of the following search terms and their abbreviations were used: psychological; stress; distress; burnout; mental health; psychiatric issues; psychological well-being; pandemic; severe acute respiratory syndrome; COVID-19, coronavirus, Ebola; influenza; H1N1; swine flu; Middle East respiratory syndrome; doctor; nurse; medical staff; healthcare worker; healthcare professional. Reference sections of included articles were scanned to identify additional studies that met inclusion criteria. Outbreaks were included if they were defined as pandemics by the WHO and included SARS (2002–2003), COVID-19 (2019–ongoing), H1N1/09 (swine flu; 2009–2010), Ebola (2014–2016), MERS (2015–ongoing) and H1N1 (influenza).

### Inclusion and exclusion criteria

Papers were included if they: (I) related to a global pandemic; (II) were written in English; and (III) investigated mediators of psychological well-being in HCWs using quantitative outcomes. Studies were excluded if they did not conduct a formal mediation analysis related to mental health outcomes in HCWs. We accepted all types of mediation analyses, including: (I) mediation analysis using the PROCESS macro extension, (II) regression with odds ratios, likelihood ratios, or other mediation analyses and (III) structural equation modelling or other path analyses.

### Data extraction and quality assessment

The first three authors independently extracted data from the identified studies. The following data were extracted: (I) author(s) and year of publication, (II) country, (III) type of pandemic, (IV) sample size and sex (percentage women), (V) age in years, (VI) profession of HCWs, (VII) study design, (VIII) measures used, (IX), type of mediation analysis, (X) mental health outcomes and mediators of mental health and (XI) study quality. For studies that described statistically significant outcomes, a *p* value < .05 was considered significant.

Quality was assessed using the Effective Public Health Practice Project (EPHPP) tool, which provides good inter-rater agreement for overall quality (Armijo-Olivo et al., 2010) across a variety of quantitative study designs (Thomas et al., 2004). Studies were assessed on: (I) selection bias, (II) study design, (III) confounders, (IV) blinding, (V) data collection methods and (VI) withdrawals and dropouts. Components were scored as 1 (‘strong’), 2 (‘moderate’), or 3 (‘weak’). EPHPP guidelines were used to generate a global score as follows: no ‘weak’ component ratings = ‘strong’, one ‘weak’ component rating = ‘moderate’ and two or more ‘weak’ component ratings = ‘weak’. The first and third author independently assessed all studies. Cohen’s kappa (Cohen, 1960) was calculated to determine inter-rater reliability, showing good agreement (94.9%) between scores (κ = .902, *p* < .001). Discrepancies were due to differences in interpretation of criteria and were discussed with the second author until a 100% agreement in coding was reached.

## Results

### Paper selection

**Figure 1.**
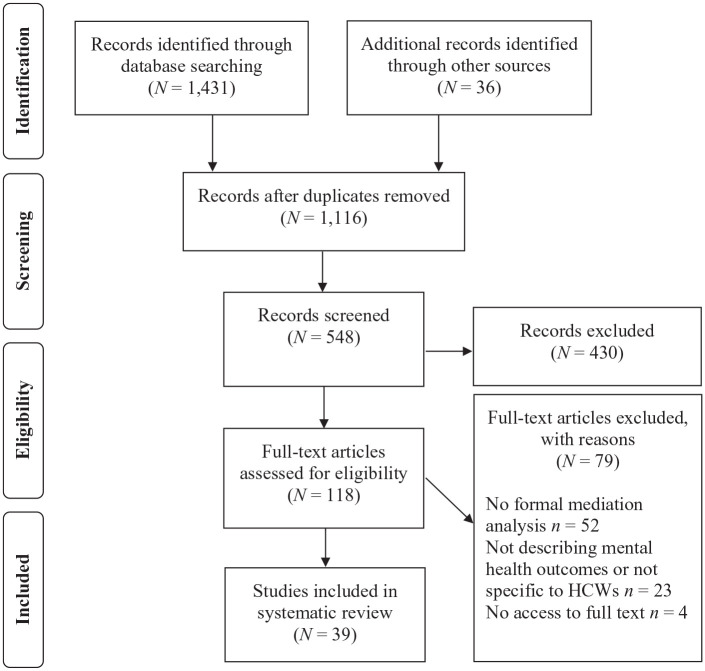
PRISMA flowchart of study selection. HCWs: healthcare workers.

As of 7 June 2020, the search protocol yielded 1467 papers (see Figure 1). After removing duplicates, 1116 papers were reviewed based on the title. Of those, 118 articles were reviewed based on the full text. Fifty-two studies were excluded because they did not conduct formal mediation analyses, 23 studies were excluded because they did not describe mental health outcomes or were not specific to HCWs, and four studies were excluded because the full text of the articles could not be accessed. All full-text articles were independently screened by the first three authors.

### Study characteristics

A final sample of 39 studies was included in this review (see Table 1), consisting of 34 cross-sectional studies and five longitudinal studies. The majority of the included studies were rated as ‘weak’ (*n* = 22) or ‘moderate’ (*n* = 15), and two studies were rated ‘strong’. Twenty-one studies investigated the SARS pandemic, twelve investigated the COVID-19 outbreak, four investigated MERS and one investigated influenza. Thirteen studies were conducted in China, eight in Canada, five in Taiwan, four in South Korea, two in Singapore and one each in India, Israel, Italy, Japan and Saudi Arabia. Most of the studies (*n* = 28) included multiple hospital staff members such as doctors, nurses, healthcare assistants, and administrative and support staff (such as cleaners and laboratory workers). Six studies focused specifically on nurses. One study was conducted exclusively on hospital doctors and one on general practitioners (GPs). In 32 studies, more than 50% of the HCWs were women. Three studies included female nurses exclusively, whereas four studies did not specify the participants’ sex. The age of HCWs ranged between 18 and 79 years, although the majority were in their twenties, thirties and forties. In terms of mediation analyses, most studies (*n* = 30) conducted multiple or logistic regression analyses (*n* = 17 with odds or likelihood ratios), seven conducted structural equation modelling, and two conducted mediation analyses using the SPSS PROCESS macro. Mental health outcomes included anxiety/worrying (*n* = 13), stress/post-traumatic stress symptoms (*n* = 16), depression (*n* = 11), sleep problems/insomnia/fatigue (*n* = 9), psychological distress (*n* = 12), fear (*n* = 3), emotional exhaustion (*n* = 3), burnout (*n* = 2), anger (*n* = 2), morbidity (*n* = 2), stigmatisation (*n* = 2), panic attacks (*n* = 1), uncertainty (*n* = 1) and obsessive-compulsive symptoms (*n* = 1).

**Table 1.** Characteristics of the reviewed studies.

Study information			Participants			Methods				Study quality
Author (year)	Country	Pandemic	*N* (% Female)	Age years M (SD)	Profession	Study design	Key measures	Mediation analysis	Mediators of psychological well-being	
Alsubaie et al. (2019)	Saudi Arabia	MERS	516 (doctors: 31%, other HCWs: 78%)	–	Doctors, nurses, technicians, respiratory therapists	CS	Anxiety	Logistic regression with ORs	Other HCWs > anxiety about contracting MERS and transmitting it to family than doctors; concern over transmitting MERS to family predicted anxiety in other HCWs	3
Amerio et al. (2020)	Italy	COVID-19	131 (48%)	52.3 (12.2)	GPs	CS	PHQ-9, GAD-7, ISI, SF-12	PROCESS macro	Anxiety and depressive symptoms mediated relationship between sleep and HRQOL (mental component)	3
Bai et al. (2004)	Taiwan	SARS	338 (52%)	39.1 (9.4)	Hospital admin personnel, HCWs, unidentified hospital staff	CS	SARS-related stress reactions	Logistic regression with ORs	Quarantine was the most related factor in development of an acute stress disorder	3
Chan and Huak (2004)	Singapore	SARS	661 (–)	60.5% 25–40 years	Doctors, nurses	CS	GHQ-28, IES, changes in life’s priorities, coping	Logistic regression with ORs	Support from supervisors and colleagues, clear communication and valuing work as important associated with decreased PTSD and psychiatric symptoms	2
Chew et al. (2020)	India, Singapore	COVID-19	906 (64%); India *n* = 426, Singapore *n* = 480	Median (IQR) = 29 (25–35 years)	Doctors, nurses, allied healthcare professionals, other HCWs	CS	Physical symptoms, DASS-21, IES-R	Logistic regression with ORs	HCWs with physical symptoms more likely to report depression, anxiety, stress and PTSD	2
Chong et al. (2004)	Taiwan	SARS	1257 (81%); initial phase *n* = 727, repair phase *n* = 530	31.8 (6.4)	Nurses, doctors, technicians, admin staff, other HCWs	L	Exposure to SARS, IES, CHQ	Logistic regression with LRs	Exposure to SARS and being in the repair phase predicted risk of psychiatric morbidity	2
Dai et al. (2020)	China	COVID-19	4357 (77%)	35.0 (8.6)	Doctors, nurses, technicians, support staff	CS	Exposure to COVID-19, risk perception, GHQ-12	Logistic regression with ORs	Identifying as female, working in Wuhan, and working in primary hospitals predicted psychological distress	2
Fiksenbaum et al. (2006)	Canada	SARS	333 (95%)	43.8 (10.0)	Nurses	CS	Contact with SARS patients and experience of quarantine, perceived SARS threat, positive feedback, SPOS, MBI, STAXI	SEM	Perceived SARS threat mediated relationship between lower perceived organisational support and emotional exhaustion and between lower perceived organisational support and state anger	3
Ho et al. (2005)	Hong Kong	SARS	97 (83%)	–	Recovered HCWs (doctors, nurses, allied health professionals, support staff)	CS	SFS, SES, IES-R	Multiple regression	SFS insecurity, SFS instability and SFS infection were significant predictors of IES-R total (48.1% variance explained)	3
Jung et al. (2020)	South Korea	MERS	147 (100%)	–	Nurses	CS	IES-R, supervisor support, turnover intention, GHQ-12, stress levels during and after outbreak	Multiple regression with covariates	Work experience 1–4 years, direct involvement with the treatment of a suspected patient, higher PTSD score and higher supervisor support (inverse) were associated with turnover intention	3
Kang et al. (2020)	China	COVID-19	994 (86%)	64.4% 25–40 years	Doctors, nurses	CS	PHQ-9, GAD-7, ISI, IES-R, exposure, accessed mental healthcare services, health status	SEM	Mental health services partially mediated the relationship between exposure risk and mental health	3
Kim and Choi (2016)	South Korea	MERS	215 (94%)	28.2 (5.5)	Nurses	CS	Burnout, job stress, fear of infection, hospital resources for treatment of MERS, support from family and friends	Multiple regression	Job stress, poor hospital resources, poor support from family and friends predicted MERS-related burnout (47.3% variance explained)	2
Koh et al. (2005)	Singapore	SARS	10511 (82%)	36.6 (11.3)	HCWs from 3 SARS and 6 SARS-free hospitals	CS	Perceived exposure, perceived risk of infection, impact on personal and work life, IES	Logistic regression with ORs	Working at a SARS hospital, being clinical staff, daily exposure to SARS patients and high IES score predicted risk perception; high IES score predicted stigmatisation; working at a SARS hospital, daily exposure to SARS patients, being a nurse, being married and high IES score predicted work stress	3
Lai et al. (2020)	China	COVID-19	1257 (77%)	64.7% 26–40 years	Doctors, nurses	CS	PHQ-9, GAD-7, ISI, IES-R	Logistic regression with ORs	Being from Wuhan and engaging in direct diagnosis, treatment and care of patients with COVID-19 were associated with a higher risk of symptoms	2
Lancee et al. (2008)	Canada	SARS	Survey *n* = 448 (86%), survey and interview *n* = 139 (87%)	Survey: 41.3 (10.2), survey and interview: 45.0 (9.6)	Nurses, other HCWs	L	IES, K10, MBI, increases in harmful behaviours, perception of adequacy of training, protection and support	Logistic regression	Previous psychiatric history, years of healthcare experience (inverse) and perception of being adequately trained or supported by hospital or clinic (inverse) predicted onset of psychiatric diagnosis after SARS	3
Liu et al. (2012)	China	SARS	549 (75%)	35% 36–45 years, 32% >45 years	Hospital employees	CS	SARS exposure, other exposure to traumatic events, perception of risk, current job stress, CES-D, IES-R	Logistic regression with mediation analyses	Quarantining, work exposure, being single, exposure to other traumatic events, perceived risk, altruistic acceptance (inverse) and high PTSD symptom level predicted higher levels of depressive symptoms	3
Lu et al. (2006)	Taiwan	SARS	127 (58%)	Doctors: 36.5 (6.3), nurses: 31.6 (5.5), other HCWs: 31.1 (7.6)	Doctors, nurses, other HCWs	CS	Impact of SARS, PBI, EPQ, CHQ	SEM	Neuroticism mediated the relationship between maternal protection and mental health symptoms	2
Lu et al. (2020)	China	COVID-19	2299 (medical staff: 78%, admin staff: 76%)	40% 31–40 years	Medical staff, admin staff	CS	Fear, HAMA, HAMD	Logistic regression with ORs	High-risk medical staff were more likely to report fear, anxiety and depression than admin staff	2
Lung et al. (2009) (follow-up of *Lu et al., 2006*)	Taiwan	SARS	127 (58%) (*n* = 123 completed follow-up)	Doctors: 36.5 (6.3), nurses: 31.6 (5.5), other HCWs: 31.1 (7.6)	Doctors, nurses, other HCWs	L	PBI, EPQ, CHQ	SEM	Neuroticism mediated the relationship between maternal protection and mental health symptoms	1
Marjanovic et al. (2007)	Canada	SARS	333 (95%)	43.8 (10.0)	Nurses	CS	MBI, STAXI, avoidance, vigour, SPOS, trust in equipment/infection control initiatives, contact with SARS patients, quarantine	Multiple regression	Contact with SARS patients, vigour (inverse) and trust in equipment/infection control initiatives (inverse) predicted emotional exhaustion (25% variance explained); time in quarantine, organisational support (inverse), vigour (inverse) and trust in equipment/infection control initiatives (inverse) predicted state anger (25% variance explained)	3
Matsuishi et al. (2012)	Japan	H1N1	1625 (76%)	30.3% 20–29 years	Doctors, nurses, other HCWs	CS	H1N1-related stress, IES	Multiple regression	Anxiety about infection higher in younger HCWs, nurses and high-risk environments; exhaustion higher in older HCWs, nurses and high-risk environments; workload stress higher in nurses and high-risk environments; feelings of being protected higher in older HCWs and nurses	3
Maunder et al. (2006)	Canada	SARS	769 (–)	–	HCWs from 9 SARS and 4 SARS-free hospitals	CS	IES, K10, MBI, increases in harmful behaviours, perception of stigma and interpersonal avoidance, adequacy of training, protection and support, job stress	Multiple regression	Maladaptive coping, perceived adequacy of training, protection and support (inverse) explained 18% of variance in burnout and 21% of variance in post-traumatic stress; maladaptive coping, attachment anxiety, experience in healthcare (inverse) explained 31% of variance in psychological distress	3
Maunder et al. (2004)	Canada	SARS	1557 (75%)	40.2 (11.0)	Hospital staff	CS	IES, attitudes towards SARS	Multiple regression with mediation analyses	Health fear, social isolation and job stress fully mediated the association of SARS patient contact and being a nurse with psychological stress (29% of variance in total IES score explained)	3
McAlonan et al. (2007)	Hong Kong	SARS	176 (73%) in 2003 and 184 (64%) in 2004	Range: 30–50 years	Doctors, nurses and healthcare assistants	L	PSS-10, DASS-21, IES-R	Multiple regression with mediation analyses	Post-traumatic stress scores partially mediated the relationship between high risk of SARS exposure and perceived stress	1
Nickell et al. (2004)	Canada	SARS	510 (–)	–	Allied healthcare professionals, non-patient-care staff, nurses, doctors	CS	GHQ-12, Occupation/work history, concerns about SARS, use and effects of precautionary measures,	Logistic regression	Being a nurse, part-time employment status, lifestyle affected by SARS outbreak and having ability to do one’s job affected by the precautionary measures predicted emotional distress	3
Park et al. (2018)	South Korea	MERS	187 (100%)	31.2 (6.8)	Nurses	CS	SF-36, PSS-10, DRS-15, stigma	PROCESS macro	The influences of stigma and hardiness on mental health were partially mediated through stress	2
Shacham et al. (2020)	Israel	COVID-19	338 (59%)	46.4 (11.2)	Dentists, dental hygienists	CS	Fear of contracting COVID-19, subjective overload, GSES, K6	Logistic regression with ORs	Background illness, fear of contracting COVID-19, subjective overload, being in a relationship (inverse) and self-efficacy (inverse) predicted psychological distress	3
Son et al. (2019)	South Korea	MERS	280 (74%)	32.4 (8.2)	HCWs and admin staff	CS	IES-R, willingness to work, coping ability, perceived risk, negative emotional experience	SEM	Negative emotional experience mediated relationship between perceived risk and willingness to work and between perceived risk and likelihood of PTSD	3
Styra et al. (2008)	Canada	SARS	248 (86%)	36.9 (9.2)	HCWs from high-risk and low-risk units	CS	Perceived personal risk, perceived risk to others, confidence in infection control measures, confidence in SARS information, impact on personal life, impact on work life, depressive affect, IES-R	Logistic regression with ORs	Working in a high-risk unit, attending only one SARS patient, perception of personal risk, impact on work life and depressive affect predicted PTSS	3
Su et al. (2007)	Taiwan	SARS	102 (100%)	Neurology: 25.4 (3.7), SARS ICU: 31.5 (6.2), regular SARS unit: 29.8 (7.6), CCU: 32.7 (4.3)	Nurses from SARS and non-SARS units	L	BDI, STAI, DTS, sleep disturbance, PSQI, attitude towards SARS, disability, family function	Logistic regression	Previous history of mood disorders predicted depressive symptoms and insomnia; age <30 and positive attitudes (inverse) predicted depressive symptoms; negative feelings towards SARS predicted PTSD symptoms and insomnia; working in SARS unit predicted sleep disturbance	2
Tam et al. (2004)	Hong Kong	SARS	652 (79%)	34.1 (8.3)	Nurses, healthcare assistants, doctors	CS	Job-related stress levels, coping behaviours, CHQ, adequacy of support systems, positive and negative perspectives of outbreak	Logistic regression with ORs	Psychological morbidity was mediated by perceptions of personal vulnerability, stress and support in the workplace	3
Verma et al. (2004)	Singapore	SARS	GPs: 721 (39%), traditional Chinese medicine practitioner: 329 (41%)	–	GPs and traditional Chinese medicine practitioners	CS	GHQ-28, IES-R, perception of stigma	Logistic regression with ORs	General practitioners: those directly involved with SARS patients more likely to score >7 on GHQ; traditional Chinese medicine practitioners: hyperarousal subscore (IES) predicted GHQ score >7	3
Wong et al. (2007) †	Canada	SARS	51 (55%)	38% <39 years; 36% 40–49 years	Doctors	CS	Training for SARS, the use of screening tools, anxiety, clinical practices	Logistic regression with ORs	Having had previous training in handling infectious disease outbreaks predicted high-anxiety classification	3
Wong et al. (2007) †	Hong Kong	SARS	137 (18%)	35.3% 40–49 years; 34.6% <39 years	Doctors	CS	Training for SARS, the use of screening tools, anxiety, clinical practices	Logistic regression with ORs	Being older, putting high value on SARS information from television, putting low value on information from the Hong Kong Medical Association Web site/circular, not losing income due to clinic closure predicted high-anxiety classification	3
Wu et al. (2009)	China	SARS	549 (77%)	47.1% 36–50 years	HCWs and admin staff	CS	SARS exposure, other exposure to traumatic events, perception of risk, IES-R, current fear of SARS	Logistic regression	Work exposure, altruistic acceptance (inverse) quarantine, and relative or friend contracting SARS predicted PTSS; risk perception partially mediated the effects of exposure on PTSS	2
Xiao et al. (2020)	China	COVID-19	180 (72%)	32.3 (4.9)	Doctors, nurses	CS	SSRS, SAS, GSES, SASR, PSQI	SEM	Anxiety, stress and self-efficacy mediated the relationship between quality of sleep and perceived social support	3
Yin et al. (2020)	China	COVID-19	371 (62%)	35.3 (9.5)	Doctors, nurses, other HCWs	CS	Exposure to COVID-19 patients, PTSD checklist, PSQI	SEM	Sleep quality fully mediated relationship between exposure level and PTSS	2
Zhang et al. (2020b)	China	COVID-19	2182 (64%)	96.3% 18–60 years	Doctors, nurses, non-medical HCWs	CS	ISI, SCL-90-R, PHQ-2, GAD-2	Logistic regression with ORs	Identifying as female, living in rural areas, exposure to COVID-19 patients and having organic diseases were risk factors for psychological symptoms in medical HCWs; having organic diseases was a risk factor for psychological symptoms in non-medical HCWs	2
Zhang et al. (2020a)	China	COVID-19	1563 (83%)	31.7% 31–40 years; 28.5% 26–30 years	Hospital staff	CS	ISI, PHQ-9, GAD, IES-R	Logistic regression with ORs	Insomnia symptoms associated with education level of high school or lower, being a doctor, currently working in isolation unit, worry about being infected, perceived lack of helpfulness in psychological support from news/social media, and having strong uncertainty regarding effective disease control	2
Zhu et al. (2020)	China	COVID-19	5062 (85%)	56.4% 30–49 years	Doctors, nurses, clinical technicians	CS	COVID-19 threat perception, IES-R, PHQ-9, GAD-7	Logistic regression with ORs	Care provided by hospital/department administrators (inverse) and protective measures (inverse) predicted stress; reasonable work shifts (inverse), support and accommodation (inverse), drinking history, and suspected/confirmed COVID-19 predicted depression; living with family members, worrying about self/family members being infected, exercise habit (inverse) and support and accommodation (inverse) predicted anxiety	2

Study quality was assessed according to the EPHPP guidelines as follows: 1 = strong, 2 = moderate, 3 = weak.

*Pandemic.* COVID-19: novel coronavirus disease 2019; H1N1: influenza; MERS: Middle East respiratory syndrome; SARS: severe acute respiratory syndrome. *Measures.* BDI: Beck Depression Inventory; CES-D: Centre for Epidemiologic Studies Depression Scale; CHQ: Chinese Health Questionnaire; DASS: Depression Anxiety Stress Scales; DRS: Dispositional Resilience Scale; DTS: Davidson Trauma Scale; EPQ: Eysenck Personality Questionnaire; GAD: Generalised Anxiety Disorder Scale; GHQ: General Health Questionnaire; GSES: General Self-Efficacy Scale; HAMA: Hamilton Anxiety Scale; HAMD: Hamilton Depression Scale; IES: Impact of Event Scale; ISI: Insomnia Severity Index; K6/K10: Kessler Psychological Distress Scale; MBI: Maslach Burnout Inventory; PBI: Parental Bonding Instrument; PHQ: Patient Health Questionnaire; PSQI: Pittsburgh Sleep Quality Index; PSS: Perceived Stress Scale; SAS: Self-rating Anxiety Scale; SASR: Stanford Acute Stress Reaction; SCL: Symptom Checklist; SES: Self-Efficacy Scale; SF-12/36: Short Form 12/36-Item Health Survey; SFS: SARS Fear Scale; SPOS: Survey of Perceived Organisational Support; SSRS: Social Support Rate Scale; STAI: Spielberger Trait Anxiety Inventory; STAXI: State-Trait Anger Expression Inventory. *Mental health outcomes.* HRQOL: health-related quality of life; PTSD: post-traumatic stress disorder; PTSS: post-traumatic stress symptoms. *Data analysis.* LR: likelihood ratio; OR: odds ratio; SEM: structural equation modelling. *Study design.* CS: cross-sectional study; L: longitudinal study. *Other.* CCU: cardiac care unit; GP: general practitioner; HCW: healthcare worker; ICU: intensive care unit; PPE: personal protective equipment.

† Split by country for clarity of results.

### Demographic mediators

Among demographic mediators, identifying as female had a greater impact on psychological distress related to an outbreak in two studies (Dai et al., 2020; Zhu et al., 2020) and was considered a risk factor for depression and anxiety in two studies (Zhang et al., 2020b; Zhu et al., 2020). Younger HCWs experienced greater anxiety (Matsuishi et al., 2012) and depressive symptoms (Su et al., 2007). Lower educational level was found to have an impact on insomnia symptomatology (Zhang et al., 2020a) and mental health more generally (Lung et al., 2009). Having an existing physical illness, long-term or otherwise, predicted psychological outcomes such as depression, anxiety, insomnia and post-traumatic stress disorder (PTSD) symptoms in three studies (Shacham et al., 2020; Zhang et al., 2020a; Zhu et al., 2020). Among HCWs, nurses experienced greater emotional distress and anxiety (Matsuishi et al., 2012) in four studies (Koh et al., 2005; Maunder et al., 2004; Nickell et al., 2004; Tam et al., 2004), although doctors were more likely to experience psychiatric and anxiety symptoms than nurses in two studies (Alsubaie et al., 2019; Chan and Huak, 2004) and reported more insomnia than other HCWs in another study (Zhang et al., 2020a). Similarly, working part-time was related with greater emotional distress (Nickell et al., 2004), as well as having fewer years of healthcare work experience (Lancee et al., 2008). HCWs’ ability to react to stress caused by pandemics was also affected negatively by history of maternal overprotection in two studies (Lu et al., 2006; Lung et al., 2009). Single HCWs were more likely to experience psychiatric symptoms such as depression compared to married participants in two studies (Chan and Huak, 2004; Liu et al., 2012), while being in a relationship was inversely related to psychological distress (Shacham et al., 2020). However, one study found that being married with children was a cause of greater stress, although the authors did not explore why (Koh et al., 2005). Finally, HCWs with physical symptoms of the pandemic disease were more likely to report depression, anxiety, stress and PTSD (Chew et al., 2020).

### Psychological mediators

Having a history of mental disorders was predictive of depression and anxiety symptomatology, insomnia, or panic attacks during the course of pandemic outbreaks in three studies (Lancee et al., 2008; Su et al., 2007; Zhu et al., 2020). Experiencing anxiety and depression during pandemics mediated the relationship between quality of sleep, quality of life (Amerio et al., 2020) and perceived support (Xiao et al., 2020). In turn, quality of sleep was influenced by exposure to the pandemic disease and to PTSD symptoms (Yin et al., 2020). Those who reported greater PTSD symptoms were also more likely to consider turnover (i.e. leave their job) (Jung et al., 2020) and to report depressive symptoms (Liu et al., 2012). Presence of PTSD during the pandemic was also associated with the degree of disease exposure and to greater levels of stress reported after 1 year from the beginning of the outbreak, especially in medical staff working in high-risk centres (McAlonan et al., 2007). Social isolation, either self-imposed or inflicted upon by others out of stigma and fear of risk of infection (Maunder et al., 2004), as well as poor support from family and friends (Kim and Choi, 2016) were related to psychological distress and risk of disease-related burnout. Feelings of self-efficacy were a protective factor from psychological distress (Shacham et al., 2020) and were increased by positive social support (Xiao et al., 2020). Xiao et al. (2020) also showed that social support was responsible for better sleep quality and reduced anxiety and stress. Moreover, altruistic acceptance of risk during the outbreak was found to decrease the odds of experiencing depressive symptomatology (Liu et al., 2012) and had a protective effect against PTSD symptoms (Wu et al., 2009). Similarly, fear of contracting SARS or infecting others, especially loved ones, was found to be predictive of anxiety and other psychological symptoms (Alsubaie et al., 2019; Ho et al., 2005).

### Organisational mediators

In ten studies, medical staff working at high-risk hospitals and in direct contact with infected patients reported greater levels of distress compared to those who were not (Dai et al., 2020; Koh et al., 2005; Lai et al., 2020; Lu et al., 2020), and experienced insomnia (Su et al., 2007; Yin et al., 2020; Zhang et al., 2020a), generally-defined psychiatric morbidity (Chong et al., 2004), post-traumatic symptoms (Wu et al., 2009), depressive symptomatology (Liu et al., 2012; Verma et al., 2004) and risk of turnover (Jung et al., 2020). In eight studies, perceived support from either the hospital, supervisors, colleagues or the government was found to protect against psychiatric disorders (Lancee et al., 2008; Tam et al., 2004), such as PTSD (Chan and Huak, 2004), depression and anxiety (Zhu et al., 2020) and avoidance behaviours and feelings of anger (Marjanovic et al., 2007). Perceived support was also related to more positive feedback on HCWs’ job performances (Fiksenbaum et al., 2006) and to reduced intentions of turnover (Jung et al., 2020) and burnout (Maunder et al., 2006). In turn, burnout was greater in the presence of job-related stress, poor hospital resources, and poor support from family and friends (Kim and Choi, 2016), as well as maladaptive coping techniques and a perceived lack of support from others, including fellow hospital staff, more generally (Maunder et al., 2006). One’s willingness to work was also affected by negative emotional experience related to the outbreak itself (e.g. fear, hurt, confusion) (Son et al., 2019). Uncertainty regarding disease control (Wong et al., 2007), being quarantined (Bai et al., 2004; Liu et al., 2012), as well as receiving poor support from the hospital (e.g. protection, workload) were related to greater disease-related stress (Fiksenbaum et al., 2006; Matsuishi et al., 2012; Shacham et al., 2020), insomnia (Zhang et al., 2020a) and burnout (Kim and Choi, 2016). Notably, news and social media also had an impact on the mental health of HCWs during global pandemics as Zhang et al. (2020b) found that lack of helpfulness and support from these sources was related to insomnia symptomatology in medical staff during the COVID-19 outbreak. Similarly, those who valued disease-related information from television, rather than formal medical organisations, were more likely to experience higher anxiety levels (Wong et al., 2007). Finally, mental health care services were considered important resources to alleviate psychological distress and protect against depression, anxiety and stress (Kang et al., 2020; Zhu et al., 2020).

## Discussion

We reviewed 39 studies in order to investigate which factors impacted the well-being of HCWs during global pandemic outbreaks. A relatively large body of studies reported that working in high-risk hospitals and/or having direct contact with suspected or infected patients was a strong risk factor for poor mental and physical health outcomes, including increased risk for anxiety, depression, PTSD, problems with sleep and lower quality of life. Most studies demonstrated that well-being was at greater risk in nurses than in other HCWs, although doctors were also found to experience an increase in stress and reduced sleep quality during pandemic events. Having symptoms of psychological illness such as depression, anxiety and PTSD as a result of the outbreaks were themselves risk factors for turnover intention, burnout and health outcomes such as disrupted sleep quality, lower perceived quality of life and greater levels of stress, thus creating the possibility for a downward cycle of increasingly poor well-being and mental health outcomes.

Other than the HCWs’ role, several studies showed that other specific demographic characteristics also mediated the relationship between various outcomes and HCWs’ psychological well-being, including identifying as female, being of a younger age, having pre-existing physical or mental health conditions, having a lower level of education and being single or unmarried. These findings are in line with previous research showing that female HCWs are more likely to experience burnout, possibly due to higher workload, poorer work-life balance and differences in work roles and responsibilities (LaFaver et al., 2018; Templeton et al., 2019). Similarly, research has suggested that younger HCWs are at higher risk of burnout than older HCWs, and that age may have a greater influence on burnout than sex (West et al., 2018). Perceived self-efficacy was shown to be a protective factor in at least one study (Shacham et al., 2020), which may explain some of these findings, as being younger, less experienced and less educated is likely to be associated with lower self-efficacy, which in turn may be associated with increased work stress and lower job satisfaction (Nielsen et al., 2009; Yao et al., 2014). In Eastern cultures, where the majority of these studies were conducted, more traditional gender roles might also mean that women were more likely to question their judgement, or have their judgement questioned, thus generating more distress (Dai et al., 2020). Being single, meanwhile, might be associated with a lack of support at home. A lack of social support from friends and family (perceived or otherwise) was an important factor in predicting poor psychological outcomes, such as burnout (Kim and Choi, 2016). Indeed, a large body of evidence suggested that social, organisational, and governmental support plays a crucial role in how global pandemic outbreaks are experienced by HCWs, and there was evidence that proper support has the potential to significantly impact their general well-being. The results of this review suggest that support was actually the most frequently reported factor for protecting HCWs’ well-being. There is, therefore, a need to ensure that support is given, especially as degradation in mental health over time can lead to perceptions of low support in the future (Xiao et al., 2020). Improving social support was also shown to increase self-efficacy (Xiao et al., 2020). Other protective factors were associated with specific personality aspects, such as altruism and ability to cope.

We found no patterns of systematic differences across pandemic type in the current review, so issues that affected HCWs during COVID-19 seemingly affected HCWs during SARS, MERS and influenza outbreaks as well. However, it is important to note that we did not conduct formal moderation analyses to investigate differences between pandemics, and more research is required for a meta-analysis of the available data. However, it is possible that culture and international differences may play a bigger role in outcome and mediator variance than type of pandemic. For example, in the United Kingdom (UK), demographic factors such as ethnicity may be important to consider, given recent evidence for Black and Minority Ethnic (BAME) inequalities across COVID-19 (Kirby, 2020). Similarly, marital status was more influential in Eastern countries (Chan and Huak, 2004; Koh et al., 2005), but did not affect the psychological well-being of HCWs in Western countries (Styra et al., 2008). Therefore, future research should explore the effects of tailored interventions that take into consideration nationality and ethnicity, though this will require more Western research into HCW’s mental health and experiences. Most studies included in this review were not longitudinal in nature, indicating that similar, rapid investigations could also be conducted across the UK, United States and Europe. At present, the majority of studies have been conducted in Asia (China, Taiwan, South Korea, Singapore, Japan and India) and North America (Canada), with two conducted in the Middle East (Israel and Saudi Arabia) and only one included study conducted in Europe (Italy). This is an important consideration when extrapolating findings from this review to other countries, where national medical systems (i.e. free healthcare in Europe) may have different expectations of, and impact on, HCWs.

Overall, the findings of this review highlight the need to focus on the mental health of HCWs before, during, and after pandemics, to promote psychological well-being and reduce adverse mental health outcomes, burnout and turnover. Below we highlight some implications of this review for policy and practice, particularly with regards to suggested targets for future interventions. Such interventions can be delivered at an individual level, by for example targeting HCWs with pre-existing physical or psychological conditions (e.g. depression) or at an organisational level, for example by providing HCWs with adequate PPE, balanced work schedules, mental health support, and appropriate accommodation and compensation.

### Policy implications and recommendations

Recent evidence suggests that ability to cope (particularly resilience) can be targeted through interventions (Chmitorz et al., 2018). Such interventions could be put in place by hospitals and other places of work. Unsurprisingly, this review found that workplaces that offered in-house mental health support served as a protective factor against depression, anxiety and stress in HCWs (Kang et al., 2020). This result, though to be expected, should reinforce the need for such services to be made available more widely. HCWs are generally reluctant to seek out mental health support even under more normal circumstances (Chew-Graham et al., 2003), but having these services in places might help with that, even if it just shows that hospitals and health workplaces are seen to be more accepting of HCWs’ mental health needs. Indeed, hospital administrators and policymakers should make efforts to ensure that nurses do not suffer from infectious disease-related stigma such as social rejection, prejudice or discrimination during the early stages of a pandemic, so that they may perceive less stress and maintain better mental health, enabling them to concentrate on caring for their patients (Park et al., 2018), even if this requires challenging accepted work cultures. Additionally, psychoeducational training (pre-pandemic) could be provided for all HCWs to help them cope with stress and negative emotions, as well as to reduce burnout. An example of such an approach is the recent application of mindfulness interventions for a variety of HCWs and healthcare settings, with promising evidence for enhanced psychological well-being outcomes (Luken and Sammons, 2016; Morgan et al., 2015; Raab, 2014). Mindfulness training can also enhance altruistic acceptance of risk among HCWs (Cameron and Fredrickson, 2015), which was found to be an important mediator of positive mental health outcomes in the current review. Similarly, the American Psychological Association (APA) has successfully piloted a psychoeducational programme to provide information about mental health to those experiencing distress, and to identify high-risk individuals who may need further intervention. Once released, the programme is planned to be available for free to psychologists and other mental health practitioners (APA, 2020). In these ways, workplaces can help ensure that HCWs perceive their job to be secure and unaffected by pandemics and other crises, as fear of turnover was a commonly reported issue (Jung et al., 2020).

Workplaces can help in other ways too. This review’s findings suggest that there is a role for providing accurate and timely information to HCWs and the public to reduce uncertainty and minimise stigmatisation of HCWs (Liu et al., 2012). Moreover, at least one study in the current review found that social media and the news cycle negatively impacted HCWs’ mental health and that having reliable and timely information from trustworthy sources was important (Wong et al., 2007), which supports recent statements on efficient leadership and ‘fake news’ (Van Bavel et al., 2020). Indeed, recent research has shown that the use of social media and exposure to COVID-19-related information through mainstream media are associated with increased levels of negative affect (Lades et al., 2020) and depression (Olagoke et al., 2020). Notably, the way mainstream media reports outbreak information is likely to differ between countries and false information and rumours are arguably easily spread online (Amin, 2020). In line with this concern, the WHO has published guidance on supporting mental and psychosocial well-being during the COVID-19 pandemic (WHO, 2020b). The guidance advises the general population to minimise the frequency of obtaining information, particularly when it causes feelings of anxiety or distress, and to seek information only from trusted sources, such as the WHO website and local health authority platforms.

Finally, it is important that healthcare organisations do not simply rely on blanket acceptance of HCWs’ duty to put their lives on the line during global pandemics. Although the contributions of volunteers and essential workers are critical during such events, social support and work safety for workers are crucial. HCWs should be aware of potential consequences of working during ongoing pandemics and be provided with the choice to withdraw from their duties, if they believe their well-being is at risk. In addition, to maintain the safety of HCWs, while also ensuring that the healthcare system can cope with increased patient cases, HCWs should be provided with compensation in the form of suitable accommodation, mental health support and social support. For the following recommendations, we must acknowledge the political, cultural, financial and other systemic factors that are likely to influence the possibility of providing suitable accommodation and the availability of other resources for HCWs. However, wherever possible, providing suitable housing to HCWs would benefit those who are concerned about the risk of infecting loved ones. Additionally, policy makers and mental health professionals working to prepare for potential disease outbreaks should be aware that the experience of being quarantined can, in some cases, lead to long-term adverse mental health consequences (Bai et al., 2004; Brooks et al., 2020; Liu et al., 2012). Similarly, perceived support has been found to be crucial and should therefore be considered a priority area for intervention. Finally, adequate PPE is an important contributor to feelings of safety among HCWs responding to infectious disease outbreaks and should be provided for all frontline workers (Simms et al., 2020).

### Strengths, limitations and future directions

Despite the rigorous search criteria and study reviews conducted, this review is not without limitations. Firstly, we found high variation among the included studies regarding outcome measures, study populations and measurement tools; thus, it was difficult to synthesise the results. Quantity of findings regarding mediators should not replace quantitative analysis of effect size through meta-analysis. At present, the quality of available studies is too limited to conduct such an analysis. Readers should be mindful that any conclusions we draw about protective and risk factors are therefore subject to scrutiny, and we encourage future research to continue better understanding the outcomes that affect HCWs’ mental health during pandemics. Secondly, the majority of studies lacked quality in study design and data collection methods. Due to the novelty of COVID-19 and other similar events that were typically investigated during the initial phase of the outbreak, many studies included unvalidated measures and failed to report the reliability of their scores, thus undermining the robustness of their findings and limiting generalisation of our conclusions. Similarly, most studies failed to properly control for confounding variables in their analyses. Assuming this was not an oversight in the analysis itself, this may well have been an issue with reporting, in which case, authors should in the future be careful not to sacrifice speed for transparency and clarity regarding the scientific process. Secondly, it is still unclear what the long-term effects of pandemic outbreaks on the mental health of HCWs are. Nearly all of the studies included in the current review were cross-sectional, with publications tending to decrease drastically after the outbreaks subsided. While data from cross-sectional studies can provide an insight into the potential mediators of mental health outcomes in HCWs, no causal inferences can be made from these observations, and longitudinal research is required to substantiate these findings. However, research interests in global pandemics seemed to dwindle as soon as the pandemic lost its novelty. Given that global pandemics are expected to become more frequent in the future (IPBES, 2020), it is crucial to increase the number of large-scale studies in order to understand which of the many variables explored so far are the most effective in increasing HCWs’ mental health in response to future outbreak situations.

Finally, there is also a need to explore the effect of pandemics on HCWs in different countries, given that available studies for the present review were conducted in few countries. Although several intervention studies are already in progress to develop and pilot mental health support packages to assist HCWs during the pandemic (e.g. Blake et al., 2020), work in this area should continue to be prioritised in order to develop multidisciplinary guidelines that may be shared at international level during the outbreak of pandemics (Zaka et al., 2020). It is important to note that, although we did not find systematic differences across pandemic type, none of the final studies included in this review examined the effects of Ebola or influenza strains outside of H1N1. It is therefore important to exercise caution before applying lessons learned from this review generally across other types of pandemics.

## Conclusions

The findings of this review are crucial to appropriately support HCWs during current and future global pandemics, as they provide up-to-date evidence on risk and protective factors that mediate the well-being of HCWs. Previously published reviews have generally focused on mental health factors exclusively or on specific outbreaks, and often failed to follow gold standard guidelines (Brooks et al., 2020; Galbraith et al., 2021), thus limiting the reliability of results and the conception of holistic interpretations. Our review is particularly relevant because it shows that individual characteristics have a significant impact on psychological outcomes during global health crises. For instance, HCWs should be aware that a history of illness may put them at higher risk of experiencing psychological symptoms and may be educated on methods of coping that are specific to their risk factors. Similarly, the well-being of those working directly with infected patients, such as nurses, should be especially monitored. The combined available evidence also shows that perceived support plays a vital role during pandemics. A safe, supporting, and efficient work environment is not only likely to impact HCWs’ well-being in various aspects of their life and work but may also benefit the hospitals. Providing appropriate training and protection to medical and administrative staff, as well as acknowledging HCWs’ need for mental care support, would reduce risk of turnover, increase medical performance in the long term, and provide positive feedback for the organisation.
